# Primary Synovial Sarcoma in the Gastric Fundus: A Case Report

**DOI:** 10.7759/cureus.24407

**Published:** 2022-04-23

**Authors:** Hosam A Alghanmi, Ammar Bokhari, Ahmed Zeeneldin, Firdos Saba

**Affiliations:** 1 Oncology Center, King Abdullah Medical City, Makkah, SAU; 2 Laboratory Department, Histopathology, King Abdullah Medical City, Makkah, SAU

**Keywords:** stomach sarcoma, case report, gastric origin, synovial sarcoma, gastric sarcoma

## Abstract

We report an unusual case of primary gastric synovial sarcoma in a young woman who presented with chronic abdominal pain. Esophagealgastricendoscopy showed a gastric fundus mass measuring 2 cm × 3 cm. Biopsy confirmed a primary synovial sarcoma. Staging work-up was negative for metastasis. The patient underwent surgery, and the histopathology results did not suggest the need for adjuvant chemotherapy.

## Introduction

Synovial sarcoma (SS) is a soft tissue sarcoma that primarily occurs in periarticular sites, but it can also develop in sites that are not related to the joints. Approximately 80% of SS cases are found in the extremities, but they have been reported in different anatomical sites other than the joints, such as the digestive system, chest, and deep visceral organs [1]. Other unusual sites include the retroperitoneum, oral cavity, and mediastinum [2]. The first reported case of SS was published in 1893 [3]. Three main histological variants of SS have been established: monophasic, biphasic, and poorly differentiated [4]. In general, most non-extremity origins of SS are case reports, and most are related to the stomach. SS has been reported in the gastrointestinal tract, including the ileum [5] and esophagus [5-7] and other parts of the digestive system, such as the liver [8] and omentum [9]. The first case of SS in the stomach was reported in 2000 [10]. Since then, many cases of SS in the stomach have been reported [1,2,11-13]. The first reported case of an unusual site of SS in Saudi Arabia was in 2012, arising from the ileum [5]. To date, there have been 40 reported cases of gastric SS, in addition to the present case.

## Case presentation

**Figure 1:**
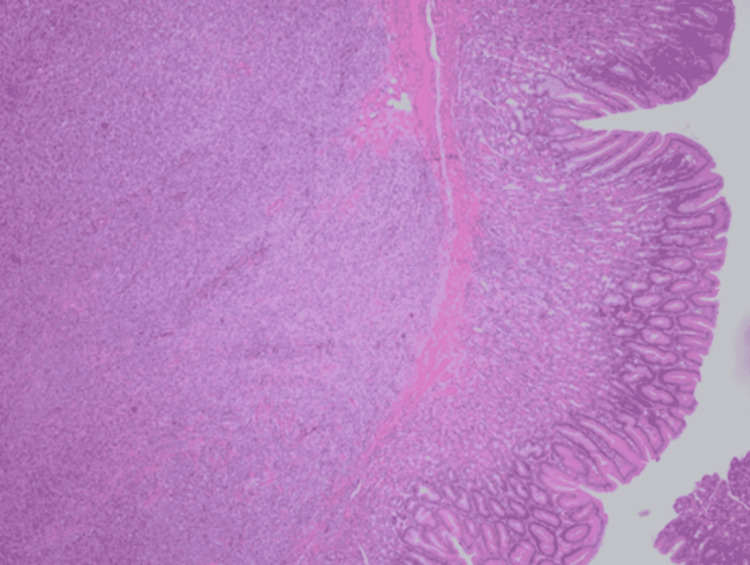
Histopathology of the gastric mucosa. A malignant tumor, present predominantly in the submucosa, is focally invading the lamina propria and extending up to the subserosa.

A 32-year-old woman without comorbid diseases presented with a history of epigastric pain that increased gradually, and decreased appetite, for two months. A systemic review ruled out any other alarming symptoms. The patient had no previous personal or family history of malignancy. On examination, there was epigastric tenderness with no masses. The other examinations were unremarkable. Laboratory investigations were within normal ranges. Because the patient’s abdominal and initial workups were negative, the patient underwent esophagogastroduodenoscopy (EGD), which showed a large polypoid ulcerative gastric mass (2 cm × 3 cm) at the anterior gastric body; multiple biopsies were taken. Histopathology revealed a spindle-cell neoplasm in the background of mild chronic focally active non-specific gastritis (Figure 1).

**Figure 2:**
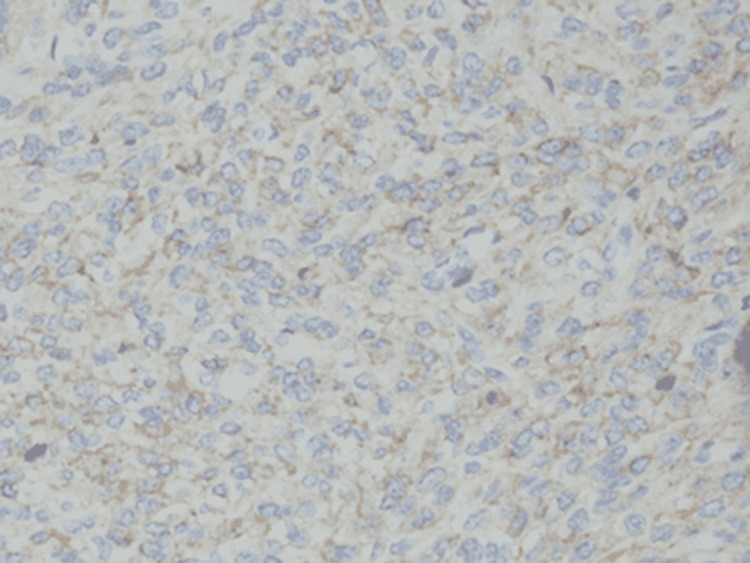
Immunohistochemistry of the EMA foci in spindle cells

**Figure 3:**
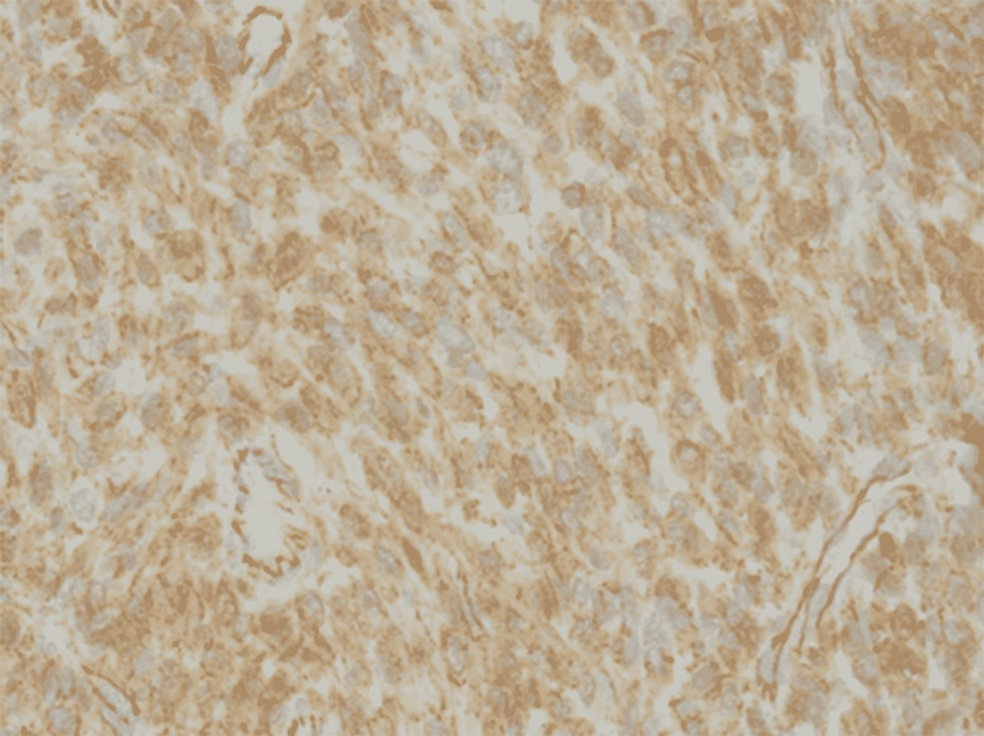
Immunohistochemistry showed spindle cells positive for nuclear and cytoplasmic β-catenin. (3)

**Figure 4:**
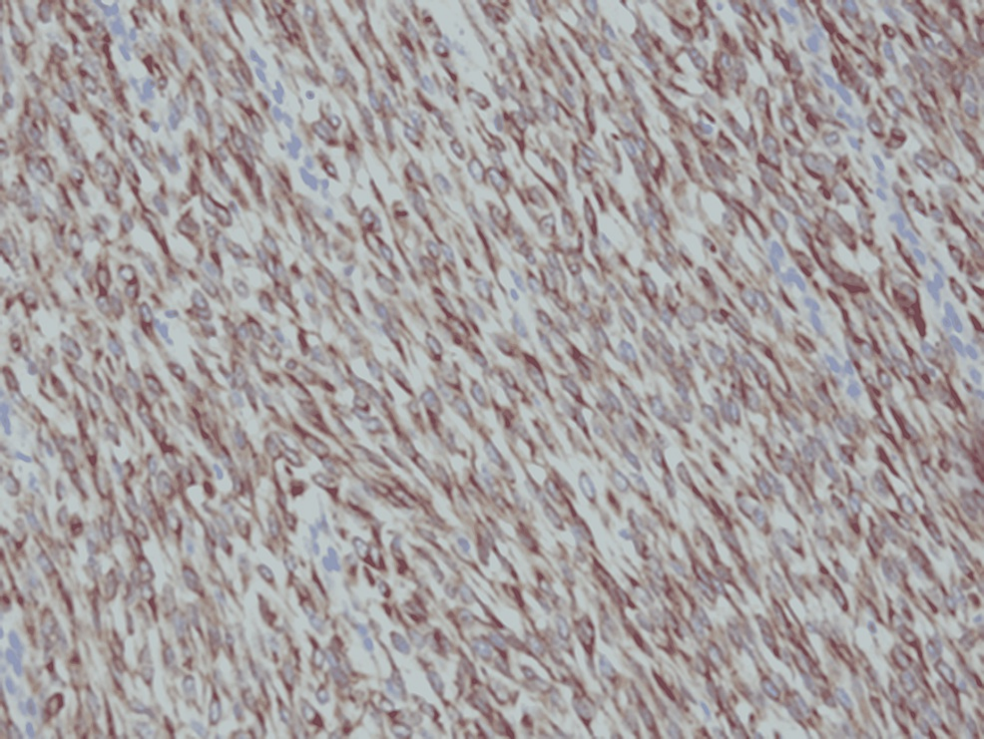
Immunohistochemistry showed spindle cells positive for BCL2

Immunohistochemistry showed weak positive EMA foci (Figure 2), strong and diffuse positive vimentin staining, positive nuclear and cytoplasmic β-catenin (Figure 3), and positive Bcl-2 staining (Figure 4).

CKAE1/AE3, CK-7, CD117, DOG1, S-100, actin SM, desmin, and CD34 stains were negative. After histopathological examination, the patient was diagnosed with malignant spindle-cell neoplasm. Further confirmatory tests of SS18F detected rearrangement of the SS18 gene region. A further metastatic workup was performed, including computed tomography of the chest, abdomen, and pelvis in August 2020, which showed focal polypoidal wall thickening at the anterior gastric body wall. No other abnormal findings were observed.

A multidisciplinary team discussion of the case in the tumor board a month later led to a plan for surgery. In November 2020, the patient underwent partial gastrectomy with an uneventful postoperative course. Histopathology revealed a 2.5 cm × 2.0 cm × 0.8 cm grade-3 tumor (tumor differentiation = 3, mitoses = 3, necrosis = 0, total score = 6) according to the French Federation of Cancer Centers Sarcoma Group. The tumor was mainly present in the submucosa and had invaded the lamina propria and muscularis propria and reached the subserosal tissue. The margins of the tumor were 0.1 cm away from the serosal surface with a negative resection margin. No perineural invasion or lymphovascular invasion was observed. No lymph nodes were identified. Therefore, this tumor was deemed stage II. The multidisciplinary team agreed on the lack of indication for adjuvant treatment. The patient is currently being followed up by imaging since November 2020 and has shown no signs of recurrence.

## Discussion

Primary gastric SS is a rare disease. To the best of our knowledge, this is the first reported case in Saudi Arabia. In a previous review of 40 cases of gastric SS, 19 cases were female, the median age was 13-67 years old, the median tumor size was 16-160 mm, the most common histopathological subtype was the monophasic type, more than 50% underwent surgical resection, and less than 25% received adjuvant chemotherapy [1]. The body of the stomach and fundus are the most common locations, but other locations, such as the gastroesophageal junction, cardia, antrum, and gastroduodenal junction, have also been reported [11].

The main prognostic factors affecting the outcome are tumor infiltration, tumor size, and histopathological subtype. Hence, a tumor size of more than 7.2 cm [11] (or more than 5 cm [2]) is associated with poor outcomes.

The different subtypes of SS resemble different types of other sarcomas. Differential diagnoses include gastrointestinal stromal tumors, leiomyoma, leiomyosarcoma, schwannoma, sarcomatoid carcinoma, and solitary fibrous tumors. Further molecular and immunohistochemical studies are needed, especially for monophasic SS [2]. SS has characteristic immunohistochemical features, such as positive staining for AE1/AE3, CK7, and EMA, but negative staining for CD117, CD34, desmin, and S100 protein [2]. A further confirmatory test by polymerase chain reaction (PCR) was used to detect the fusion of the SYT-SSX genes [14].

As reported previously, the main treatment step is surgical resection, either by wedge resection or total gastrectomy. The role of chemotherapy is limited, and only a few patients have received neoadjuvant chemotherapy [1,2,11]. The treatment outcome is excellent, and fewer patients need adjuvant treatment due to the effectiveness of surgical management.

No standardized follow-up program is required for such cases; hence, surveillance by clinical, radiological, and endoscopic means is recommended.

In our case, the patient underwent partial gastrectomy due to the small tumor size. Histopathology suggested a very early tumor that did not require adjuvant chemotherapy or radiation. Follow-up imaging after surgery showed no recurrence or metastasis. Since discharge, the patient has been under follow-up with imaging since 2020.

## Conclusions

Primary gastric SS is extremely rare and difficult to diagnose. There are no clear guidelines for this type of tumor. Treatment is based on previous reports and some retrospective studies. The diagnosis of such cases requires advanced immunohistochemistry and advanced molecular studies. Multidisciplinary team management is essential to provide the needed treatment. The main line of treatment is radical surgery. The role of neoadjuvant or adjuvant chemotherapy is limited. More studies are needed to standardize the treatment steps in these cases. Medical and surgical oncology reports on such cases, with sharing of management experience, should be encouraged.
